# Hippocampal proton MR spectroscopy as a novel approach in the assessment of radiation injury and the correlation to neurocognitive function impairment: initial experiences

**DOI:** 10.1186/s13014-015-0518-1

**Published:** 2015-10-17

**Authors:** Petr Pospisil, Tomas Kazda, Martin Bulik, Marie Dobiaskova, Petr Burkon, Ludmila Hynkova, Pavel Slampa, Radim Jancalek

**Affiliations:** Department of Radiation Oncology, Faculty of Medicine, Masaryk University, Kamenice 5, 625 00, Brno, Czech Republic; Department of Radiation Oncology, Masaryk Memorial Cancer Institute, Zluty kopec 7, 656 53, Brno, Czech Republic; International Clinical Research Center, St. Anne’s University Hospital Brno, Pekarska 53, 656 91, Brno, Czech Republic; Department of Diagnostic Imaging, Faculty of Medicine, Masaryk University, Kamenice 5, 625 00, Brno, Czech Republic; Department of Diagnostic Imaging, St. Anne’s University Hospital Brno, Pekarska 53, 656 91, Brno, Czech Republic; Department of Clinical Psychology, St. Anne’s University Hospital Brno, Pekarska 53, Brno, Czech Republic; Department of Neurosurgery - St. Anne’s University Hospital Brno, Faculty of Medicine, Masaryk University, Kamenice 5, 625 00, Brno, Czech Republic; Department of Neurosurgery, St. Anne’s University Hospital Brno, Pekarska 53, 656 91, Brno, Czech Republic

**Keywords:** Hippocampus, Radiation injury, Neurocognitive function, Magnetic resonance spectroscopy

## Abstract

**Background:**

The hippocampus is considered as the main radiosensitive brain structure responsible for postradiotherapy cognitive decline. We prospectively assessed correlation of memory change to hippocampal N-acetylaspartate (h-tNAA) concentration, a neuronal density and viability marker, by ^1^H-MR spectroscopy focused on the hippocampus.

**Methods:**

Patients with brain metastases underwent whole brain radiotherapy (WBRT) to a dose of 30 Gy in ten fractions daily. Pre-radiotherapy ^1^H-MR spectroscopy focused on the h-tNAA concentration and memory testing was performed. Memory was evaluated by Auditory Verbal Learning Test (AVLT) and Brief Visuospatial Memory Test-Revised (BVMT-R). Total recall, recognition and delayed recall were reported. The both investigation procedures were repeated 4 months after WBRT and the h-tNAA and memory changes were correlated.

**Results:**

Of the 20 patients, ten passed whole protocol. The h-tNAA concentration significantly decreased from pre-WBRT 8.9, 8.86 and 8.88 [mM] in the right, left and both hippocampi to 7.16, 7.65 and 7.4 after WBRT, respectively. In the memory tests a significant decrease was observed in AVLT total-recall, BVMT-R total-recall and BVMT-R delayed-recall. Weak to moderate correlations were observed between left h-tNAA and AVLT recognition and all BVMT-R subtests and between the right h-tNAA and AVLT total-recall.

**Conclusions:**

A significant decrease in h-tNAA after WBRT was proven by ^1^H-MR spectroscopy as a feasible method for the in vivo investigation of radiation injury. Continuing patient recruitment focusing on other cognitive tests and metabolites is needed.

## Background

Although the general improvement in the current management of cancer patients has led to an increase in the overall survival rate, more and more patients develop brain metastases (BM) [1]. Whole brain radiotherapy (WBRT) is a basic therapeutic approach used for its treatment. WBRT is, however, associated with adverse side effects leading to the possible worsening of the quality of life, particularly in relation to the worsening of neurocognitive function [2, 3]. Current clinical studies are providing increasing evidence of an association between a hippocampal radiotherapy (RT) dose and cognitive impairment [4, 5, 6]. Recently, new strategies have been investigated in order to minimize these adverse effects, particularly for patients with favorable prognostic factors [4, 7, 8]. One promising approach, enabling the preservation of neurocognitive functions (NCF) is hippocampal sparing during WBRT, as recently proven by phase II study [4] and currently being investigated in the ongoing phase III clinical trials (NCT01942980, NCT01780675, NRG CC001, NRG CC1432). However, further basic research is still necessary in order to provide a deeper insight into processes responsible for the hippocampal radiation injury.

Proton magnetic resonance spectroscopy (MRS) is a diagnostic and research method enabling an in-vivo examination of the spatial distribution of specific tissue metabolites concentration. Total N-acetylaspartate in the hippocampus (h-tNAA), including N-acetylaspartate together with N-acetylaspartylglutamate, is one commonly detectable brain tissue metabolite that is considered to be the marker of neuronal density and viability [9]. Although the process of hippocampal radiation injury is multifactorial [10], neuronal depletion by apoptosis is considered to be an essential process leading to NCF impairment [11, 12]. A detailed investigation of the metabolic response of hippocampus to irradiation reflecting possible neuronal depletion is still lacking.

Herein, with the first ten analyzable patients, we present our initial interim analysis providing an evaluation of the correlation between the h-tNAA concentration dynamics and the changes in NCF impairment in patients after WBRT. The primary endpoint of the study was to evaluate the post-WBRT decrease of the h-tNAA concentration measured by MRS. The secondary endpoint was the correlation between the h-tNAA concentration decrease and changes in memory function.

## Methods

### Patient selection

Patients with a current history of cancer disease and with either newly diagnosed BM or immediate postoperative radiotherapy after the surgical resection of a single metastasis that were referred for WBRT in the Department of Radiation Oncology, Faculty of Medicine, Masaryk University and Masaryk Memorial Cancer Institute between May 2013 and September 2014 were considered for study enrollment. Patients met inclusion criteria when having Karnofsky performance status ≥ 70 % and favorable survival prognosis of 4 months as predicted by a graded prognostic assessment [13] (index ≥ 1.5 is needed for prediction of survival higher than 3.8 months; age, Karnofsky status, number of brain metastases and presence of extracranial metasetases are used for index calculation). Patients with worse survival prediction and those suffering from other neurological or psychiatric diseases or patients with radiologic pathology in the hippocampus region found during pretreatment MRI were excluded. The study was approved by the Institutional Review Board and all of the patients provided their written informed consent before study enrollment.

### MRS examination

A single slice multi-voxel spectroscopic examination was performed using GE Medical Systems Discovery MR 750 3 T (PRESS-CSI sequence with TE/TR =135 ms/1690 ms, 12 averages, FOV 120 × 120 mm^2^) at the Department of Diagnostic Imaging, St. Anne’s University Hospital Brno. The region of interest was placed through whole temporal lobi with the voxel layer position adjusted based on the localization of hippocampi in order to examine the whole hippocampi at long distance (voxel size set to 10 × 10 × 15 mm^3^) [14]. Postprocessing of raw spectroscopic data was performed using the LCModel [15] for the calculation of the h-tNAA absolute concentrate [mM] which were further visualized and analyzed by java Spectroscopic Imaging PROcessing software (jSIPRO) [16] for final reporting of the mean h-tNAA concentration. The final selection of voxels of interest was partially automated. In the first step, MRS voxels where hippocampus represented more that 2/3 of the covered tissue were manually selected and those with a spectral error value of greater than 20 % were subsequently automatically excluded. This error takes into account signal to noise ratio and full width at half peak maximum as proposed by Jiru et al. [17]. During the whole process of voxels selection, final metabolite concentrations were not visible ensuring blinding of analysis. By using jSIPRO software for reporting the calculated MR spectroscopic maps, it is possible to select all voxels within both hippocampi at an overlaid axial T2-weighted image. On average, nine voxels were analyzed per right and left hippocampus. The variability in the number of voxels available for analysis was mainly due to exclusion of some voxels because of low quality of spectral data (high error value). MRS was performed prior to WBRT and repeated 4 months later using the same methodology.

### Neurocognitive function evaluation

NCF were examined by experienced psychologists at the maximum time interval of five working days around MRS. Standardized tests focusing on memory were assessed: AVLT (Auditory Verbal Learning Test) and BVMT-R (Brief Visuospatial Memory Test - Revised). The AVLT includes memorizing 15 words for five consecutive attempts (Total Recall, TR), recalling them after 30 min (Delayed Recall, DR) and subsequently identifying these words from a list of related words (Recognition, R). During the follow up examination, a standardized retest variation of words was used. The BVMT-R includes memorizing six geometric figures for three consecutive attempts (TR) and similarly as with the AVLT recalling them after 25 min (DR), and finally identifying them among those offered in the list of related figures (R). Standardized alternate form of BVMT-R was used for follow up examination.

### Radiotherapy

WBRT was delivered by standard external beam radiotherapy based on two opposing laterolateral equally weighted fields; the multi-leaf collimator was used to shape the fields in such a way that the whole brain was included (prescribed dose to the isocenter located in the middle of brain). Treatment was delivered with six megavoltage photon beams of linear accelerator. Patients were treated in a head-first supine position with the head immobilized by individually prepared thermoplastic masks. RT beams were defined in an RTG 2D simulator (Varian Acuity iX) to homogenously cover the whole brain while shielding the facial tissue. The prescribed dose was uniform in all patients: 30 Gy in ten fractions delivered in 2 weeks.

### Data analysis

Obtained data were compared on a case-by-case basis where each patient was his or her own control. The relative decline in the h-tNAA concentrations was expressed as Δtest = (test control – test baseline) ÷ test baseline. For NCF tests, absolute score changes were reported. Standard descriptive statistics were applied in the analysis; absolute and relative frequencies for categorical variables and mean supplemented by standard deviation and median with min-max range for quantitative variables. Wilcoxon’s signed rank test was adopted for the computation of the statistical significance of differences in paired quantitative data. The relationship between quantitative variables was analyzed using the Spearman correlation coefficient and its statistical significance. The follow-up was calculated as the period from the date of the BM diagnosis to the death of the patient or the last contact with the patient; the overall survival time was analyzed using the Kaplan-Meier methodology. Statistical analysis was performed using JMP 10 Software (SAS Institute) and two-sided α = 0.05 was taken as a level of statistical significance in all analyses.

## Results

### Patient’s characteristics

**Fig. 1:**
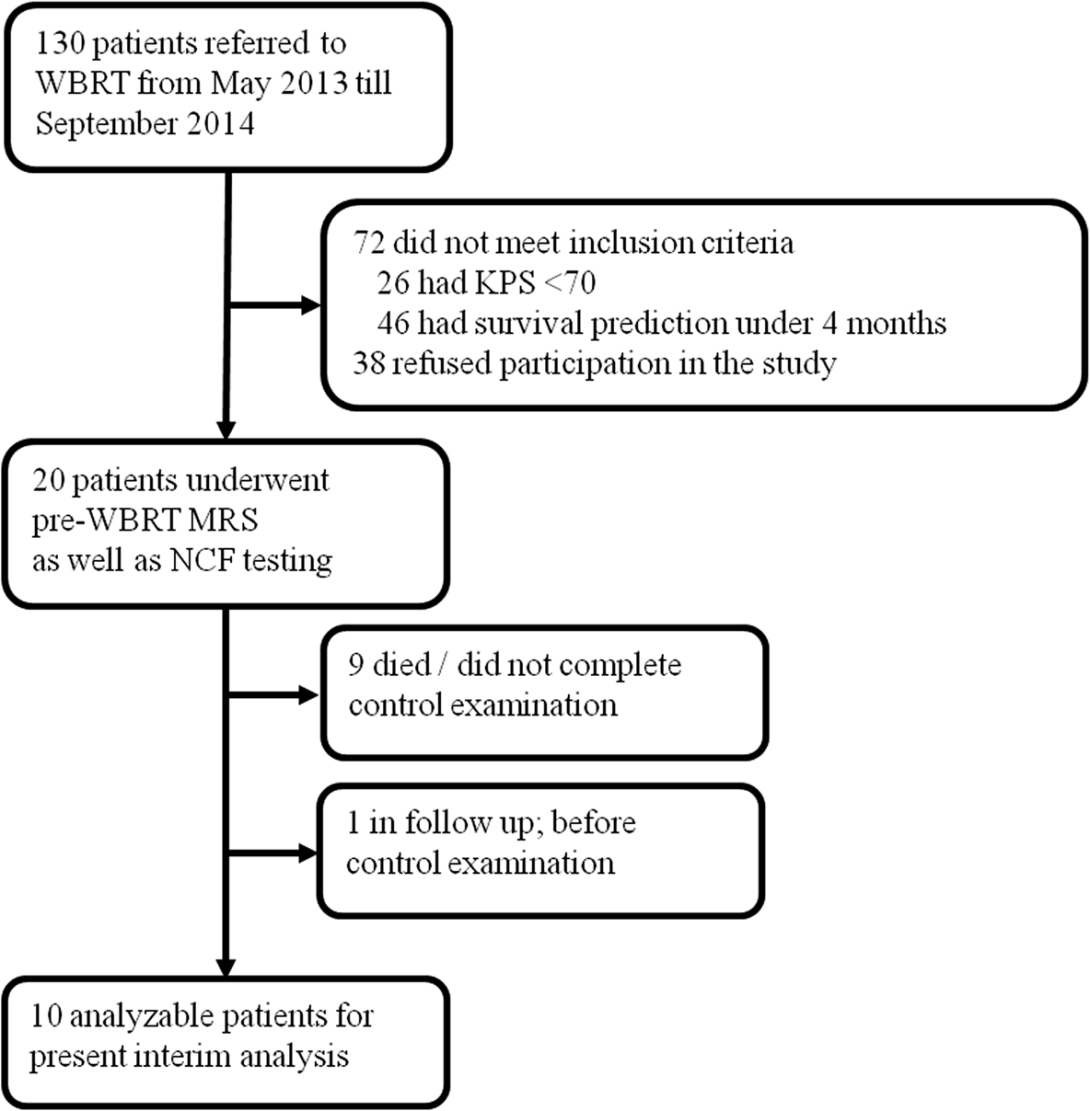
The flow diagram of patient enrolment

A total of 20 patients with a median age of 60 years and a median 90 % Karnofsky performance status met the inclusion criteria. The majority of patients had lung cancer (six patients) and the median number of metastases was 1 (1–20). Ten (50 %) patients had already completed the planned control examination 4 months after the WBRT and were further analyzed (mean time to control 4.3 months ± 0.7 months). Their characteristics are summarized in Table 1. One (5 %) patient is still to take the control examination and nine (45 %) died or did not comply with the control examination (Fig. 1). The median overall survival was 11.9 months (95 % CI 7.5 to 13.9 months) for patients who underwent control examinations after 4 months and 9.2 months (95 % CI 3.5 to 13.9 months) for all 20 included patients.

**Table 1:** Basic characteristics of ten analyzable patients

Patients characteristic					Pre-WBRT			MTS			Post-WBRT CHT	Time relapse to end of WBRT	
No	Sex	Age	Hand	Tumor	KPS	GPA [mo]	Surg	No	Location	Volume [ccm]		B [days]	F/U [mo]
1	M	57	r	NSCLC	90	6.9	Yes	2	r_F(1) + T(1)	4.2	Yes	16	5.1
2	M	53	r	RCC	90	3.8	No	1	r_P(1)	0.4	Yes	23	4.6
3	W	63	r	Breast	80	3.8	No	5	r_F(1) + P(2) + O(1)	0.1	Yes	17	3.4
									l_pons(1)				
4	M	66	r	Occult	90	3.8	Yes	2	r_P(1), l_O(1)	0.05	Yes	34	3.7
5	W	47	r	Breast	90	3.8	No	20	r_F(3) + P(1) + O(1)	15.5	Yes	20	3.5
									l_F(6) + P(3) + T(1) + O(3) + Crbl(2)				
6	M	69	r	GI	90	3.8	Yes	1	l_Crbl(1)	0	Yes	21	4.7
7	M	65	r	GI	90	6.9	Yes	1	l_F(1) + P(1)	0	No	17	3.4
8	M	63	r	RCC	90	6.9	No	1	r_Crbl(1)	0.1	Yes	17	4.7
9	W	58	r	Ovarian	100	11	Yes	1	r_Crbl(1)	0	No	24	4.6
10	W	48	r	Cervical	90	6.9	No	1	l_Crbl(1)	3.7	No	21	4.8

*No* number, *M* men, *W* women, *Dg* diagnosis, *NSCLC* non–small-cell lung cancer, *RCC* renal cell cancer, *GI* gastrointestinal cancer, *KPS* Karnofsky performance status, *MTS* metastases, *GPA* Graded Prognostic Assessment [13], *mo* months, *Surg* surgery, *r* right, *l* left, *F* frontal, *T* temporal, *P* parietal, *O* occipital, *Crbl* cerebellum, *CHT* chemotherapy, *B* baseline examination, *F/U* follow-up examination. Location: the number of metastases is mentioned in brackets

### Hippocampal MR spectroscopy

**Fig. 2:**
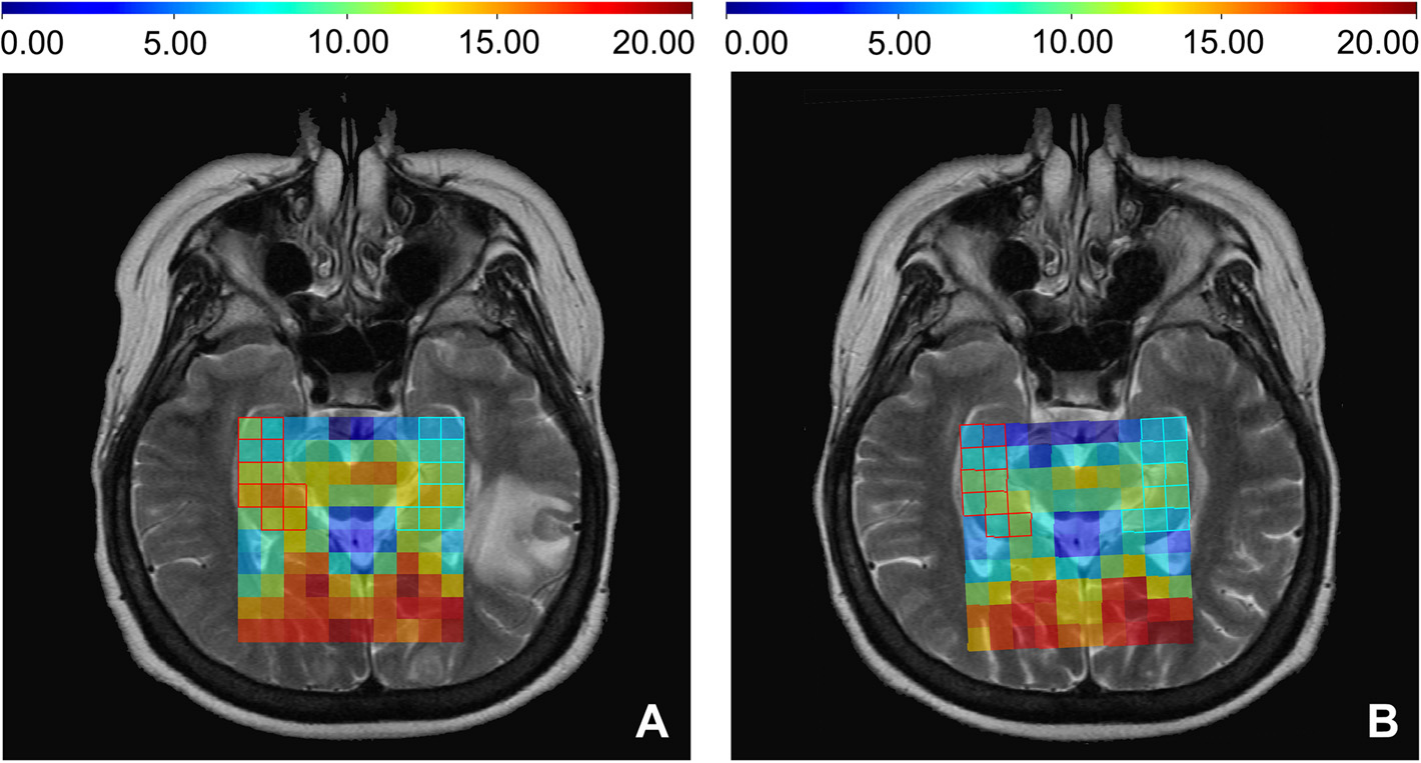
Pre-WBRT (**a**) concentration of h-tNAA in the patient number 5 (11.29 mM in the right and 10.55 mM in the left hippocampus) and its decrease after WBRT (**b**) (8.14 mM in the right and 8.42 in the left hippocampus)

A post-WBRT decrease in the average h-tNAA concentration was consistent in all ten analyzable patients with minimal–5 % (patient number 1) and maximal–25 % (patient number 9) decrease (Table 2). Figure 2 displays pre-WBRT concentration of h-tNAA in the patient number five and its remarkable decrease after WBRT. No difference was observed in the h-tNAA concentration between the right and left hemisphere.

**Table 2:** Mean post-WBRT relative declines of the h-tNAA concentration and absolute changes in memory tests

No	h-tNAA			AVLT			BVMT-R		
	RH	LH	BH	TR	DR	R	TR	DR	R
1	−7 %	−3 %	−5 %	−12	−3	0	−19	−6	0
2	−14 %	−10 %	−12 %	−3	0	0	−2	−2	−1
3	−14 %	−19 %	−17 %	−16	−2	0	−8	−4	0
4	1 %	−14 %	−6 %	−11	+1	+2	−5	−1	−1
5	−28 %	−20 %	−24 %	−3	−2	+3	−4	−2	+1
6	−22 %	−21 %	−22 %	−10	−5	+1	0	−2	−1
7	−26 %	1 %	−13 %	−2	0	−1	−8	−3	−1
8	−27 %	−2 %	−15 %	−14	0	0	−4	−4	−3
9	−29 %	−22 %	−25 %	0	+2	−1	−18	−5	−1
10	−15 %	−22 %	−19 %	−13	−3	+3	0	+2	0

*No* patient’s number, *h-tNAA* total N-acetylaspartate in the hippocampus, *RH* right hippocampus, *LH* left hippocampus, *BH* both hippocampi, *AVLT* Auditory Verbal Learning Test, *BVMT-R* Brief Visuospatial Memory Test-Revised, *TR* total recall, *DR* delayed recall, *R* recognition

### Neurocognitive function analysis

All ten analyzable patients completed all of the tasks in the AVLT and BVMT-R tests. The relative declines in all tasks are summarized in Table 2 together with relative declines in the h-tNAA concentrations calculated using the same equation.

In our group of patients in the AVLT_TR, the mean score decline (−8.4 points) was statistically significant in the control examination compared to pre-WBRT tests (*p* = 0.0039). In the corresponding BVMT-R_TR a significant decline was also observed (−6.8 points; *p* = 0.008). Moreover, the decline was also ascertained in the BVMT-R_DR (−2.7 points; *p* = 0.001). Mean differences between the baseline and control examination are summarized in Table 3.

**Table 3:** Absolute mean differences between pre-WBRT and post-WBRT examination for the h-tNAA concentrations [mM] and all AVLT and BVMT-R subtests

	Pre-WBRT	Post-WBRT	Absolute mean difference		Relative mean difference [%]	
			(95 % CI)	*p*-value^+^	(95 % CI)	*p*-value^‡^
h-tNAA	[mM]	[mM]				
RH	8.9	7.16	−1.74	0.004*	−18.1	0.004*
					(−11 to –25.2)	
			(−0.99 to −2.48)			
LH	8.86	7.65	−1.21	0.004*	−13.4	0.004*
					(−6.9 to −19.9)	
			(−0.56 to −1.86)			
BH	8.88	7.401	−1.48	0.002*	−15.9	0.002*
					(−10.9 to −20.9)	
			(−0.92 to −2.04)			
AVLT						
TR	45.1	36.7	−8.4	0.004*		
			(−4.3 to −12.5)			
DR	7.3	6.1	−1.2	0.125		
			(0.33 to −2.7)			
R	12.8	13.5	0.7	0.250		
			(1.77 to −3.69)			
BVMT-R						
TR	22.6	15.8	−6.8	0.008*		
			(−1.96 to −11.6)			
DR	9.2	6.5	−2.7	0.001*		
			(−1.1 to −4.3)			
R	5.6	4.9	−0.7	0.109		
			(0.06 to −1.45)			

*CI* confidence interval, Asterisks denote statistical significance; cross denotes Wilcoxon signed test; double-cross denotes Wilcoxon signed rank test, hypothesized value 0; *h-tNAA* total N-acetylaspartate in the hippocampus, *RH* right hippocampus, *LH* left hippocampus, *BH* both hippocampi, *AVLT* Auditory Verbal Learning Test, *BVMT-R* Brief Visuospatial Memory Test-Revised, *TR* total recall, *DR* delayed recall, *R* recognition, *WBRT* whole brain radiotherapy

No statistically significant strong correlation was observed between the decrease in the right, left and overall h-tNAA concentration and the decrease in the AVLT and BVMT-R subtests scores.

Weak positive correlation was observed between left h-tNAA concentration and AVLT_DR (Spearman correlation *r* = 0.24, *p* = 0.5). Weak to moderated negative correlation was observed between left h-tNAA and AVLT_R (*r* = −0.47, *p* = 0.17), BVMT-R_TR (*r* = −0.36, *p* = 0.30), BVMT-R_DR (*r* = −0.36, *p* = 0.30) and BVMT-R_R (*r* = −0.39, *p* = 0.27). For right h-tNAA concentration, the negative correlation was only to AVLT_TR (*r* = −0.5, *p* = 0.14).

## Discussion

The personalized approach in medical care is increasingly discussed also in the management of patients suffering from BM [18, 19]. Preserving neurocognitive functions and the quality of life is becoming an important target in clinical trials as well as in daily practice, especially after WBRT [20, 21]. Since even low dose radiation injury to the neural stem subgranular zone cells of the hippocampal dentate gyrus is related to early cognitive and memory decline [22], hippocampal sparing during WBRT seems to be the most promising approach [4, 23, 24] alongside pharmacological interventions [7, 25, 26]. Ongoing research which further ascertains processes responsible for hippocampal radiation injury may provide additional evidence supporting a particular personalized approach as well as revealing new strategies for mitigating the adverse neurocognitive effects of WBRT. However, additional factors, such as tumor-related morbidity, as well as the effects of surgery and chemotherapy may also contribute to final NCF impairment and must be taken into account.

In the present study, the post-WBRT neurocognitive decline was investigated in correlation to an innovative hippocampal examination by proton MR spectroscopy. To the best of our knowledge, this is the first in human study documenting the cognitive decline related to post-WBRT hippocampal metabolic changes as proven by noninvasive in vivo examination. Using hippocampal MRS is well established in cognitive disorder research particularly focusing on mild cognitive impairment (MCI), an early stage of dementia [27, 28]. NAA was found to be the most reliable marker of brain cognitive and memory dysfunction and MRS is presumed to be the predictor of the progression of MCI into Alzheimer dementia [29] as well as a predictor of the conversion of cognitively normal older adults into MCI [30].

Proton MR spectroscopy of the hippocampal region was also performed for the examination of postradiation metabolic changes in the brain, but only with limited regional differences in specific hippocampal evaluation [31–37]. The NAA reduction after radiotherapy was consistent throughout all mentioned studies, however, direct comparisons may be biased due to inconsistency in the use of the spectroscopy method (single voxel, single slice multi-voxel, 3D echo planar spectroscopic imaging), target voxels placement, patient selection (primary or secondary brain tumors, therapeutic or prophylactic brain irradiation) as well as due to a lack of cognitive assessment with the exception of the Mini-Mental Status Examination evaluation in the Movsas et al. study [33], where no correlation to whole-brain decrease in NAA was observed 3–4 weeks after WBRT.

The feasibility of human quantitative spectroscopic measurement of radiation induced hippocampal brain injury was proven by investigators from the University of Pennsylvania who examined h-tNAA, creatine and choline as well as diffusion tensor imaging in seven patients 1 month after WBRT using 3D echo planar spectroscopic imaging. A trend towards a decrease in the ration of NAA and creatine was observed from the hippocampal region 1 month after radiation (1.48 ± 0.07 vs 1.27 ± 0.2, *p* = 0.06) [37]. In our study, a significant decrease of the h-tNAA spectra was observed in the both hippocampi as well as separately in the left and right hippocampus (single slice multi-voxel spectroscopic examination).

Functional magnetic resonance imaging can demonstrate the functional anatomy of cognitive and memory processes. Functional memory asymmetry is known mainly from epileptology where mapping the sites of memory function is important before neurosurgical planning for temporal lobe epilepsy. Left hippocampus is more related to verbal memory function comparing to visual memory connected more to the right one [38]. Similar asymmetry can be expected also in our cohort, since all analyzable patients were right handed. Although weak (*r* = 0.24), positive correlation was observed between decrease of left h-tNAA concentration and decrease in the AVLT_DR absolute score. This correlation may confirm the hypothesis that WBRT leads mainly to damage of relatively mitotically active neuronal stem cells which results in lower ability to maintain verbal memories.

The negative correlations between h-tNAA and NCF evaluated by both AVLT and BVMT-R (for example moderate negative correlation (*r* = −0,5) with trend to statistical significance (*p* = 0,14) between right h-tNAA and AVLT_TR) are probably biased by small sample size as well as selection bias of our patient cohort. However, consistent decrease in NAA concentrations and NCF test were observed in all patients. More analyzed patients are needed to provide some conclusions.

Altogether, it is too early to draw any clear conclusion with unequivocal explanation of observed correlations considering also potential effects of pre-WBRT neurocognitive dysfunction due to primary tumor-, patient- or chemotherapy-related effects or even gender [39, 40]. Indeed, some patients had relatively severe NCF decrease as proved by low scores in memory tests which may impact severity of post-WBRT changes. Nevertheless, results of presented interim analysis warrant continuing requirement in our ongoing study with other secondary analyses as evaluation of absolute NAA concentrations or hippocampal volumetry analyses. With more included patients, the true correlation may be discovered. The main advantage of the proposed research methodology is the non-invasive nature of the examination represented by advanced MR imaging which may be easily added to standard diagnostic imaging protocol. It may be assumed, that similar research may enhance pre-radiotherapy imaging description of hippocampal function in individual patient and guide asymmetric hippocampal avoiding WBRT just as preoperative functional MRI has potential to predict postoperative verbal memory decline after anterior temporal lobe resection [41].

This small prospective clinical investigative study has numerous limitations. The Hopkins Verbal Learning Test - Revised (HVLT-R) is currently probably the most reliable test for the evaluation of radiation induced cognitive impairment [42]. However, proficiency in English is required and so its standardized Czech version was used in our study, which prevents a direct comparison with cited seminal randomized trials. The other limitation is seen in a narrowly focused MRS as well as NCF evaluation in this initial experience report. More metabolites (choline) as well as normalized concentrations with respect to creatine are among the most meaningful candidates for extended patient’s requirements as well as for a retrospective analysis of already included patients.

## Conclusion

A significant decrease in h-tNAA after WBRT was proven by ^1^H-MR spectroscopy as a feasible method for the in vivo investigation of radiation injury. To definitely assess whether hippocampal avoiding RT approaches are worth the increased cost and effort of their performance, patients and tumor related biomarkers need to be established for the proper selection of suitable patients. Advanced MRI methods have the potential for the description of early adverse effects of brain irradiation long before standard white matter postradiation changes are visible with the time delay being beyond the average survival time of patients with BM. Studies similar to ours, where potential imaging biomarkers are correlated to prospectively evaluated neurocognitive changes, may identify which biomarker best correlates to the final affected treatment outcome and end point, i.e. an improvement in the quality of life of patients treated with palliative intent. Our promising results support continuing recruitment in ongoing studies focusing on other NCF tests as well as hippocampal metabolites.

## Abbreviations

AVLT: Auditory verbal learning test
BM: Brain metastasis
BVMT-R: Brief visuospatial memory test-revised
DR: Delayed recall
h-tNAA: Total N-acetylaspartate in the hippocampus
HVLT-R: Hopkins verbal learning test-revised
MCI: Mild cognitive impairment
MR: Magnetic resonance
MRS: Magnetic resonance spectroscopy
NCF: Neurocognitive functions
R: Recognition
RT: Radiotherapy
TR: Total recall
WBRT: Whole brain radiotherapy
